# Humanism in Medicine: A Cardiac Patient’s Perspective

**DOI:** 10.1016/j.jaccas.2024.102560

**Published:** 2024-09-18

**Authors:** Cecilia R. Varga

**Affiliations:** University of Florida College of Medicine, Gainesville, Florida, USA

**Keywords:** arrhythmia, cardiac ablation, cardiology, electrophysiology, humanism

As a competitive athlete, I never anticipated having a heart condition. Nonetheless, supraventricular tachycardia (SVT), a benign and treatable arrhythmia, seemed to be the best possible outcome after years of struggling with undiagnosed symptoms. No one could have predicted the debilitating years of progressive, drug-refractory arrhythmias that followed my initial diagnosis, necessitating 9 electrophysiology studies (EPSs), 7 of which involved ablations and, ultimately, the loss of my ability to compete in a sport that was my greatest passion. Although arduous, my journey has resulted in a deeper and more intimate understanding of human suffering that allows me to better care for my patients.

In an occupation highly susceptible to human error, compounded by challenging internal and external factors within the health care system, the humbling reality is that we sometimes fall short in how we care for patients. Regardless of intentionality, our words and actions can have a lasting impact. We should seek to learn from the experiences of our patients and, in doing so, improve how we care for them. Although revealing aspects of my journey is daunting, the prospect of positively changing the narrative for other patients and igniting an essential discussion on humanism within our profession is worth the vulnerability of sharing my story.

At 16 years of age, I was lying beside my indoor rowing machine and desperately trying to maintain consciousness. Accustomed to pushing my physical limits, I was familiar with a pounding heart after a physically demanding workout. However, my heart was beating so rapidly that it was hardly countable. When these primarily exercise-induced episodes of tachycardia continued to occur, my long quest for a diagnosis began.

Despite numerous consultations, my condition went undiagnosed for 3 years, and I was repeatedly met with dismissive remarks from physicians, who suggested my symptoms were the result of a psychiatric disorder. When I finally received the diagnosis of SVT, I was relieved to have an answer. However, years of repeated trivialization left an indelible imprint on me; lingering self-doubt and a fear of unnecessarily burdening people, including my doctors, consistently affected my ability to advocate for myself.

My diagnostic challenge is not uncommon, especially in women. A significant association exists between gender and the frequency of misdiagnosis, severity of symptoms, and duration until ablation.^1^, ^2^, ^3^ Furthermore, increased levels of anxiety and mistrust during interactions with future medical professionals, as well as uncertainty and self-doubt, are common among patients whose conditions are misdiagnosed or who are invalidated during the diagnostic process.^4^ Although I recognize that the diagnosis of rhythm disturbances depends on electrocardiographic evidence, a discussion that sets realistic expectations and includes compassionate listening and validation can help facilitate greater trust and understanding, thus attenuating the psychosocial consequences of a prolonged diagnostic process.

Once I had a diagnosis of SVT, multiple medications helped control my episodes. However, I eventually became unresponsive to medication and underwent my first EPS in pursuit of a cure. Described as a safe and minimally invasive procedure with a high success rate, an EPS with ablation seemed promising. In hindsight, my desire to return to my collegiate rowing team impaired my judgment that day. Eighteen years old and medically naïve, I did not question the electrophysiologist, who assured me that an anesthesia provider was not necessary, even on standby and that I would only receive lidocaine. Six years later, vivid memories remain, including excruciating pain during an unexpected medication reaction and the embarrassment of being left unclothed in restraints on the procedure table. Perhaps an oversight, a precautionary intravenous line was never placed, and the team was not prepared to respond to a medication reaction. I still remember being overwhelmed with fear as I begged for help while suffering in pain and slowly losing consciousness. This procedure did not change the arrhythmia, but the brutal, dehumanizing experience forever changed me.

My condition progressed beyond the scope of conventional medicine in the ensuing years. I trialed a dozen combinations of antiarrhythmic medications and underwent multiple invasive substrate ablations, yet I remained debilitated by frequent, daily arrhythmia episodes with heart rates as high as 290 beats/min. The metabolic demand was so profound that I lost 20 pounds within a few months. Desperately longing for a reprieve, I consulted numerous electrophysiologists. However, I often struggled to express the gravity of the emotional and physical toll because the one time I had shown vulnerability in the past, a physician told me that my situation paled in comparison with someone with cancer and I had no reason to cry. In consideration for a spot on the United States Rowing Team, but without a viable solution, I watched my identity as a powerful athlete dissipate as I struggled to walk up a flight of stairs. I fought with everything I had to maintain my athleticism, but in the end, that dream was not meant for me.

My experience as an arrhythmia patient has been the most challenging battle of my life. Yet I am grateful for the lessons and perspective I gained through it. No matter how much medical education emphasizes the patient experience, no one can truly understand it until illness falls on them. Accordingly, I feel compelled to share a few lessons I have learned.

First, there is power in our words and actions— as much as they can offer healing, they can also cause significant harm. Moreover, there is power in listening. Sometimes, validating a personal struggle by simply listening intently can offer as much healing power as treatment. The memories that remain with me occurred in mundane moments at the bedside—we must always be mindful of the lasting impact we can have during patient interactions. There is a difference between a patient being well cared for and feeling well cared for. The latter, dependent on the subtle art of humanism, is often underappreciated but can be profoundly impactful.

Second, although a condition may not be life-threatening, that does not mean it is not life-altering. Although some patients have vastly more devastating illnesses than others, the severity of the disease should not dictate the extent of compassion and understanding we show patients. Furthermore, I urge caution in using the word *benign* to describe certain arrhythmias, especially to athletes. I used the word benign to justify pushing through my symptoms. My tenacious mindset was my greatest strength as an athlete but also my greatest liability as a patient because I often found myself in unsafe situations, one of which resulted in a concussion.

Third, there is a tremendous need for patience and empathy in the electrophysiology laboratory. Being physically restrained on a table, unable to move or see anything, while catheters manipulate the heart, is uncomfortable and frightening. Small acts of compassion, even simply checking in on the patient or communicating before pacing maneuvers, are easy ways to humanize the experience. Moreover, before performing an ablation, a simple inquiry about past experiences can illuminate areas to improve and personalize care and foster trust in the physician (eg, tell me about your past procedures; what can we do to make you feel most comfortable?). Patients may not voluntarily share their honest thoughts, especially if they had previous negative experiences. Therefore, inquiry invites an open discussion.

Lastly, the psychological implications of a fully conscious ablation should be carefully considered in the decision-making process. Although anesthesia can negatively affect arrhythmia inducibility, lack of anesthesia does not guarantee successful induction. We should seek merciful options that balance inducibility with patient comfort. A compassionate and mindful approach is critical if a decision is made to proceed with minimal or no sedation. In cases where the patient is awake, we must remember that they can hear and feel everything, and if they are aware enough, they will likely remember it all.

Although my journey has been a heartbreaking challenge, I believe our response to trials in life is a conscious choice. We can use adversity as an excuse to harden us or as a motivation to improve. The same concept applies to the daily challenges faced by medical professionals. Humanism in medicine is more important now than ever, and its revival depends on each of us making a conscious choice to enact change and learn from our patients, whose stories have a pivotal role in transforming how we care for them. Caring for and comforting people who invite us into their most intimate, darkest moments are humbling privileges. May we choose to be a light.

## Funding Support and Author Disclosures

The author has reported that she has no relationships relevant to the contents of this paper to disclose.
